# Intermetallic Compound and Solid Solutions of Co75Me25 (Me: Si, Fe, Cr) as Catalysts for the Electrochemical Reaction of Nitrate Conversion to Ammonia

**DOI:** 10.3390/ijms26041650

**Published:** 2025-02-14

**Authors:** Irina Kuznetsova, Dmitry Kultin, Olga Lebedeva, Sergey Nesterenko, Elena Murashova, Leonid Kustov

**Affiliations:** 1 Department of Chemistry, Lomonosov Moscow State University, Moscow 119991, Russia; kuznetsowair@yandex.ru (I.K.); lebedeva@general.chem.msu.ru (O.L.); snnest@gmail.com (S.N.); murashovaev@rambler.ru (E.M.); lmk@ioc.ac.ru (L.K.); 2 N.D. Zelinsky Institute of Organic Chemistry, Russian Academy of Sciences, Leninsky Prospect 47, Moscow 119991, Russia

**Keywords:** intermetallic compounds, synthesis of ammonia, nitrate reduction, electrocatalysis, sustainable chemistry, energy chemistry, intermetallic catalyst, Co catalyst, bimetallic catalyst

## Abstract

A sustainable reaction of electrocatalytic nitrate conversion in ammonia production (NO_3_RR) occurring under ambient conditions is currently of prime interest, as well as urgent research due to the real potential replacement of the environmentally unfavorable Haber–Bosch process. Herein, a series of electrocatalysts based on two-component cobalt alloys was synthesized using low-cost non-noble metals Co, Fe, Cr, and also Si. The samples of electrocatalysts were characterized and studied by the following methods: SEM, EDX, XRD (both transmission and reflection), UV–VIS spectroscopy, optical microscopy, linear (and cyclic) voltammetry, chronoamperometry, and electrochemical impedance spectroscopy. Beyond that, the determination of electrochemically active surface area was also carried out for all samples of electrocatalysts. Unexpectedly, the sample having an intermetallic compound (IMC) of the composition Co_2_Si turned out to be the most highly effective. The highest Faradaic efficiency (FE) of 80.8% at E = −0.585 V (RHE) and an ammonia yield rate of 22.3 µmol h^−1^ cm^−2^ at E = −0.685 V (RHE) indicate the progressive role of IMC as the main active component of the electrocatalyst. Thus, this study demonstrates the promise and enormous potential of IMC as the main component of highly efficient electrocatalysts for NO_3_RR. This work can serve primarily as a starting point for future studies of electrocatalytic conversion reactions in the production of ammonia using IMC catalysts containing non-noble metals.

## 1. Introduction

The sustainable electrocatalytic reaction of nitrate conversion in ammonia production (NO_3_RR) occurring under ambient conditions using renewable energy sources is currently of prime interest as a promising potential alternative to the environmentally unfavorable Haber–Bosch process and simultaneous treatment of nitrogen-containing pollutants [1,2]. Optimization of reactors and reaction conditions together with the inventive design of new electrode materials and electrolytes serve to provide better electrocatalytic performance and control of reaction conditions [3,4,5].

While the attention of researchers is focused on the search for an effective electrocatalyst using two-metal systems, such as bimetallic catalysts [6] or heterostructures (oxides [7], sulfides [8], and phosphides [9] of two metals), or single-atom catalysis (SAC), intermetallic compounds (IMCs) remain virtually beyond the attention of modern and recent works.

Intermetallic compounds are considered as a new class of advanced electrocatalysts among binary catalysts [10]. Diverse electronic structures of IMC and long-range ordered atomic arrays with constant stoichiometry and well-defined crystal structure determine catalytic properties that differ from the properties of an alloy (solid solution) having a random atomic arrangement with the same composition. Ordered atomic arrays enhance catalytic activity by increasing the number of surface sites that have an optimal binding strength to reaction intermediates. Theoretical calculations have shown that the ordered arrangement of atoms in the intermetallic phase enhances the ligand effect, neatly tuning the electronic structure of the catalytic surfaces. However, the effect of the well-defined morphology on catalysis is not usually discussed in detail.

It should be noted that high-entropy IMCs are potentially promising materials for electrocatalysts, combining the advantages of ordered IMCs and the multicomponent nature of high-entropy alloys [11,12]. The use of such IMCs for catalysis requires size reduction from bulk to nanoscale. The increased number of components and harsh annealing conditions inevitably cause agglomeration. Larger nanoparticles have a larger thermodynamic barrier to the disorder/order phase transition, which leads to the formation of IMCs, as well as phase separation. Strong ionic/covalent interactions between metal components are expected to enhance the stability of intermetallic catalysts during the catalytic process in comparison with monometallic analogues and alloys. Intermetallic compounds, due to their ordered crystal structures, can effectively regulate the adsorption energies of catalytic reaction intermediates through coordination/strain effects, thereby optimizing the catalytic activity.

Typical industrial electrocatalysts mainly use noble metals, so the search for their efficient replacement is focused on base metals, for example, cobalt due to its high reactivity, such as Pt_3_Co/CoNC nanostructures [13]. IMCs based on d-metals find applications in energy conversion reactions for *fuel-cells*, for *hydrogen evolution reaction* and *water splitting* [14,15,16]. Compared to other binary catalysts, IMC as NO_3_RR electrocatalysts have been studied to a lesser extent [2,17,18,19,20].

The adsorption energies of three key intermediates (*NO_3_^−^, *NO, and *H_2_O) for 21 types of transition-metal-based intermetallic compounds and Sb/Bi (IMCs) as electrocatalysts were compared using density functional theory (DFT) [2]. The results show that the hexagonal CoSb IMC possesses the optimal adsorption equilibrium for the key intermediates and experimentally demonstrated outstanding electrocatalytic performance in NO_3_RR with the Faradaic efficiency (FE) of 96.3% and NH_3_ selectivity of 89.1%, as well as excellent stability.

PdCu intermetallic nanocubes were used as a catalyst for the reduction of nitrate to NH_3_, showing a high FE of 92.5% [17], while intermetallic single-atom alloy In-Pd, in which Pd atoms are isolated by In atoms, showed an FE of 87.2% [18].

A comparison of methods of IMC preparation and their characterization and activity in NO_3_RR reactions, as well as in other known and demanded electrochemical reactions (nitrogen, nitrite, and NO reduction) was presented in the review [19]. A nanoporous intermetallic single-atom alloy CuZn catalyst with fully isolated Cu-Zn active centers exhibits FE above 95% over a wide potential range from −0.2 to −0.8 V (RHE) [20]. The explanation for the high catalytic activity was based on the strong electronic interactions between isolated Cu and Zn active centers that alter the protonation of adsorbed particles, effectively reducing the Gibbs energy of intermediate protonation of *NO_2_ as the rate-determining step. Another sustainable and green reaction is co-reduction of CO_2_ and nitrate reduction leading to electrochemical synthesis of urea on IMC-alloy AuCu thin films grown electrochemically onto Ti foil substrates and showing enhanced selectivity and activity compared to the parent metals [21].

A multi-strategy approach based on the development of ordered mesoporous intermetallic AuCu_3_ with an ultrathin Au shell as an efficient and concentration-insensitive catalyst for NO_3_RR was considered [22]. Such a novel IMC catalyst exhibits an outstanding FE exceeding 95%. The study [23] substantiates that structurally ordered intermetallic nanocrystals and single-atom catalysts are two new kinds of catalysts for sustainable chemical production and energy conversion. However, both materials have synthetic limitations that can lead to aggregation of particles or metal atoms. The authors were able to synthesize a Cu/CuAu IMC, which has a high selectivity towards NH_3_ in NO_3_RR with an FE of 85.5%.

A thermal processing strategy has been proposed to obtain an antiperovskite with regular and ordered Ga-Cu coordination [24], which binds via strong p-d orbital-oriented hybridization to balance the catalytic activity and crystal stability. As a result, the highly ordered Ga-Cu_3_N catalyst provides an impressive FE of 96.48%. The rapid formation of active hydrogen (*H) and low energy barrier on the facets of Ga-Cu IMC are confirmed by theoretical calculations. The nanoporous intermetallic phase Al_11_Ce_3_ was used as an example to achieve an EF of 91% [25]. A general method for the preparation of a series of Ga transition-metal-based IMCs, including Co-Ga, Ni-Ga, Pt-Ga, Pd-Ga, and Rh-Ga IMCs, is presented here [26]. Structurally ordered CoGa IMCs with the bulk-centered cubic structure are uniformly distributed on a reduced graphene oxide substrate.

It was reported [27] that the IMC of the Cu_2_TiSn composition demonstrates high nitrate reduction rates with FE equal to 77.14% and maintains high stability for 100 h under neutral conditions.

An original idea to revise the prevailing mechanistic picture of nitrate hydrogenation on bimetallic ordered PdCu catalysts is expressed. What is essential is the electrical pathway for the current flow from Pd to Cu and the ionic flux of proton equivalents in solution, rather than any interaction at the atomic level between the two constituents. In their work [28], the authors note that interactions at the atomic level can benefit NO_3_RR catalysts in certain cases, but well-ordered intermetallic PdCu catalysts with a large number of bimetallic active centers may work via a special mechanism.

Bimetallic Co-Fe electrocatalysts [6], as well as CoSb IMC [2] showed prospects for their use in the NO_3_RR reaction in a neutral medium. Some IMCs, such as Ni_2_Si, NiSi, or NiSi_2_, show higher activity compared to nickel, which was explained [29] by the strong interaction between nickel and silicon, preventing sintering of nanoparticles.

The aim of this work was to test the efficiency of the electrocatalyst with the main component in the form of IMC of base metals (both components) for the NO_3_RR reaction. The following tasks were accomplished: samples of Co75Me25 (Me: Si, Fe, Cr) alloys were synthesized; the alloys were characterized by SEM, EDX, XRD, and optical microscopy, and the IMC was detected; optimum conditions were found by electrochemical methods, and NO_3_RR measurements were performed; and electrochemically active surface area and charge transfer resistance were determined.

## 2. Results and Discussion

### 2.1. Structure and Composition Characterization of Electrocatalysts

#### 2.1.1. SEM and EDX Analysis

**Figure 1:**
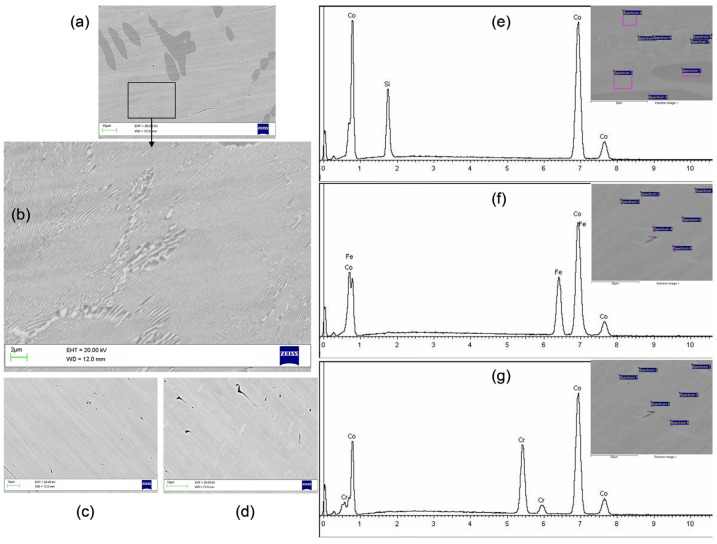
SEM images: (**a**,**b**) Co75Si25 (eutectic alloy); (**c**) Co75Fe25 (solid solution); (**d**) Co75Cr25 (solid solution). EDX elements spectra for (**e**) Co75Si25 (eutectic alloy); (**f**) Co75Fe25 (solid solution); (**g**) Co75Cr25 (solid solution). Insertion on (**e**–**g**) demonstrates various locations for the EDX probe.

Scanning electron microscopy images of the studied electrocatalysts are shown in Figure 1. The surface of the Co75Si25 sample (Figure 1a) consisted of two clearly visible phases, and the second phase was especially clearly visible in the enlarged image of Figure 1b in the form of scaly waves. Two other samples of electrocatalysts, Co75Fe25 and Co75Cr25, did not have a clearly defined relief or other phases on their surfaces (Figure 1c,d). The EDX analysis for each sample is shown in Figure 1e–g and indicates a composition containing Co as the main component and, respectively, Si, Fe, or Cr as the second, additional component. Alloy Co75Si25 is an eutectic alloy consisting of two phases—Co_2_Si and a solid solution of Co75Si25. Alloys Co75Fe25 and Co75Cr25 are single-phase materials. The EDX probe data indicate the sample compositions shown in Table 1.

These results show that the Co75Si25 alloy consists of two phases of different compositions, and one of them belongs to the intermetallic compound Co_2_Si. The presence of Co, which has a catalytic effect in NO_3_RR in the intermetallic state, is of ***fundamental*** importance for this work. The authors believe that this can enhance the electrocatalytic properties of Co even more and to a significant extent. To clarify the composition and structure, X-ray analysis was performed in Section 2.1.2.

#### 2.1.2. X-Ray Diffraction Analysis

**Figure 2:**
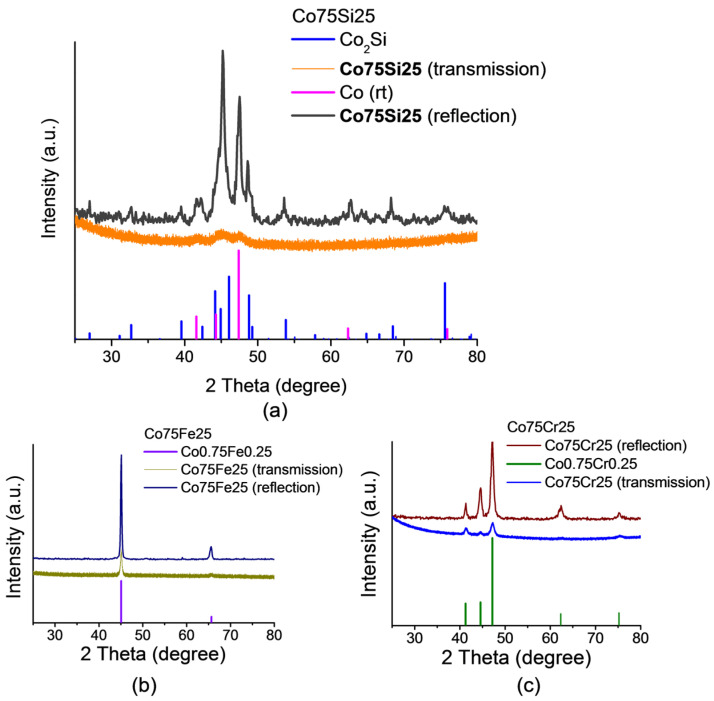
XRD patterns for electrocatalyst samples: (**a**) Co75Si25 (eutectic alloy); (**b**) Co75Fe25 (solid solution); (**c**) Co75Cr25 (solid solution). Pictures for both transmission and reflection of X-rays are given.

X-ray diffraction powder patterns of all three samples (Co75Si25, Co75Fe25, and Co75Cr25) are shown in Figure 2a–c. Reflections on the X-ray diffraction powder patterns of the studied samples show low intensity due to anomalous X-ray scattering, since the wavelength of the radiation (CuKa1) was located near the edge of the absorption band of the elements (Co) present in the samples. Experimental X-ray data for all samples were obtained in the “reflection” geometry, and additional research was conducted in the “transmission” geometry for samples Co75Si25 and Co75Fe25. Reflections with high-intensity lines are clearly visible in X-ray diffraction patterns, which make it possible to determine the qualitative composition of the studied alloys using the JCPDs X-ray database: 71-7109 (Co0.75Cr0.25), 71-7173 (Co0.75Fe0.25), 71-7435 (Co (rt)), 89-4181 (Co_2_Si). The reflections in the geometry “for transmission” were less intense, but their positions coincided with the positions of the corresponding reflections of the X-ray pattern measured in the “reflection” mode.

According to X-ray phase analysis, the Co75Si25 sample is a two-phase material; one of the phases is the intermetallic compound Co_2_Si, the second one is a low-temperature Co phase. The Co-Fe and Co-Cr samples are solid solutions of the compositions Co75Fe25 and Co75Cr25 with a cobalt structure. The XRD results on the composition of the obtained phases are in good agreement with the compositions shown in Section 2.1.1 and in Table 1.

#### 2.1.3. Confirmation by Optical Microscopic Images

**Figure 3:**
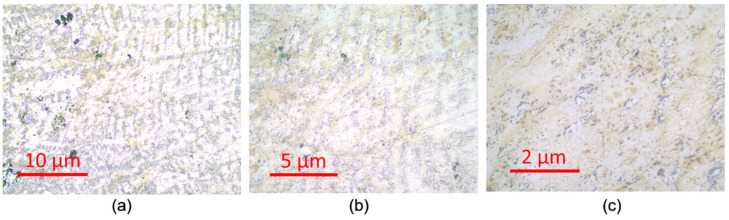
Optical images of the Co75Si25 sample containing the eutectic phase at different magnifications: (**a**) 200×; (**b**) 500×; (**c**) 1000×.

Optical microscope images are shown in Figure 3a–c for the Co75Si25 sample containing the eutectic phase. The tree-like structure of the second IMC phase is clearly visible in the general solid solution phase. The images for Co75Fe25 and Co75Cr25 are more homogeneous and represent a single phase of a solid solution.

### 2.2. Electrochemical Synthesis of Ammonia via the NO_3_RR Reaction

#### 2.2.1. Linear Voltammetry Studies

**Figure 4:**
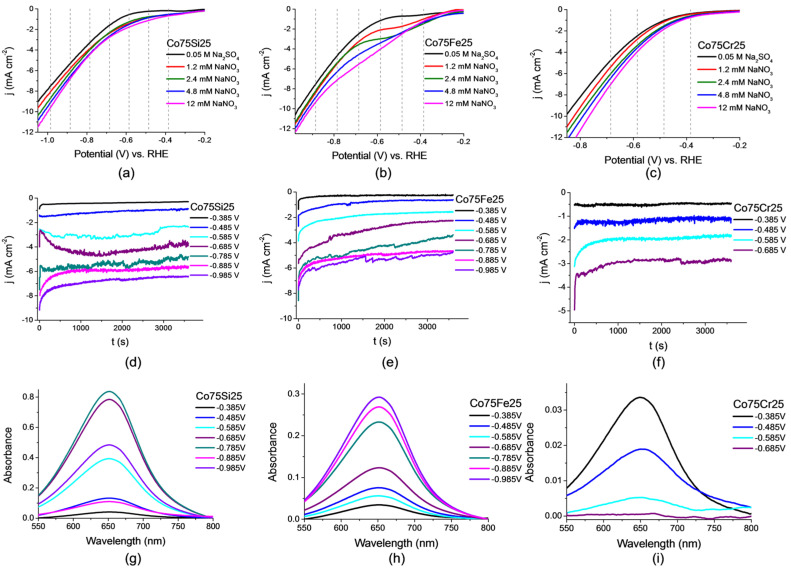
Linear voltammetric curves in the 0.05 M Na_2_SO_4_ electrolyte at different concentrations of nitrate ions at a potential scan rate of 50 mV s^−1^ for electrocatalyst samples: (**a**) Co75Si25 (eutectic alloy); (**b**) Co75Fe25 (solid solution); (**c**) Co75Cr25 (solid solution). The process of ammonia synthesis by NO_3_RR (chronoamperometric curves in the 0.05 M Na_2_SO_4_ + 12 mM NaNO_3_ electrolyte) at different potentials for (**d**) Co75Si25 (eutectic alloy); (**e**) Co75Fe25 (solid solution); (**f**) Co75Cr25 (solid solution). UV–VIS spectrum corresponding to the concentrations of the resulting product formed via NO_3_RR at λ = 652 nm: (**g**) Co75Si25 (eutectic alloy); (**h**) Co75Fe25 (solid solution); (**i**) Co75Cr25 (solid solution).

Voltammetric measurements were performed to identify the optimal potential for the synthesis of ammonia by NO_3_RR. Linear voltammograms (LVs) were obtained in the potential range E = −0.2 V to −1.0 V, that is, from the non-Faradaic region of the double electric layer, in which there are no processes in the absence of the observed current, to almost the region of molecular hydrogen release via the HER reaction. As can be seen from Figure 4a–c, the rise in the current density for all three samples of catalyst electrodes begins approximately at a potential of E = 0.35 V and continues up to the boundary of the selected interval.

With an increase in the nitrate concentration, an increase in the current rise was observed, which indicates active reduction reactions occurring with the participation of nitrate ions. The exception, obviously, was a nitrate-free electrolyte, where the current density rise began in a much more cathodic region, almost at a potential of E = −0.65 V for Co75Si25 and Co75Fe25 and at a potential of E = −0.55 V for Co75Cr25, which indicates the absence of reactions associated with the reduction of nitrate ions at these potentials.

A further increase in the nitrate concentration was not advisable for two reasons: firstly, with an increase in the nitrate concentration, incomplete nitrogen reduction and synthesis processes begin to prevail [30], and secondly, the use of high concentrations does not simulate solutions typical for wastewater (where the concentration is not too high) [31]. LV curve graphs do not contain obvious peaks, plateaus, or waves associated with a selective process, so we are talking about choosing a potential range for NO_3_RR. The optimal interval for obtaining high ammonia yield rates at high FE, therefore, from our point of view is E = −0.5 V to −0.8 V.

#### 2.2.2. Chronoamperometric Measurements and Nitrate Conversion

To confirm the conclusions drawn on the basis of the LV research results, the values of synthesis potentials for NO_3_RR were selected, and a series of reactions were carried out for 1 h. The selected potentials are indicated by dotted lines in Figure 4a–c and correspond to the values E = −0.385; −0.485; −0.585; −0.685; −0.785; −0.885, and −0.985 V. The NO_3_RR kinetics were close to zero at lower potential values, and, at higher ones, the side reaction of hydrogen evolution (HER) started to significantly dominate.

Chronoamperograms (or electrolysis at a controlled potential) for all three samples of catalyst electrodes are shown in Figure 4d–f. It is clearly noticeable that the average current density for the selected potential values here was usually higher for the sample Co75Si25 (eutectic alloy) than for Co75Fe25 (solid solution) and even higher than for Co75Cr25 (solid solution). It is obvious to assume that the Co75Si25 electrocatalyst is preferable for the main NO_3_RR process, although it can be expected from the literature data that the almost bimetallic Co75Fe25 catalyst should exhibit the greatest activity. We discuss this effect in more detail at the end of this section in “A brief summary of the elucidation for the proposed mechanism of electrocatalysis”.

It should be noted that the NO_3_RR reaction time should be sufficient for the spectrophotometric determination of ammonia, and it was chosen in accordance with recent research by the authors of [6,32] and with the literature data of other recent works. The UV–VIS spectra corresponding to the concentration of ammonia synthesized in the NO_3_RR process for each sample of the electrocatalyst are shown in Figure 4g–i. As can be seen from the figures, the ammonia concentration maxima were observed at different potential values for all samples. The Co75Si25 sample demonstrated the optimal option, where the maximum was found in the middle of the selected potential range (E = −0.785 V). In the case of the other two samples, the picture was completely different: Co75Fe25 (E = −0.985 V) and Co75Cr25 (E = −0.385 V) provided the maximum concentration of the product at a potential, which either favored the competing HER reaction or was insufficient to provide fast reaction kinetics, respectively.

#### 2.2.3. Analysis of Faradaic Efficiency and Ammonia Yield Rate

**Figure 5:**
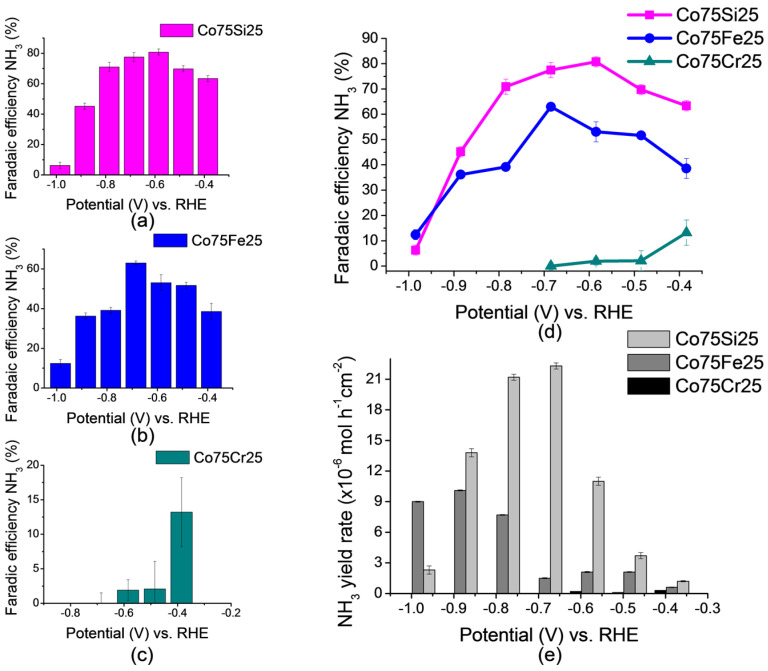
The results of the NO_3_RR study: (**a**) FE values for Co75Si25, (**b**) FE values for Co75Fe25, (**c**) FE values for Co75Cr25, (**d**) the resulting general FE-graph, and (**e**) the ammonia yield rate of NO_3_RR.

Based on the results of chronoamperometric measurements, FE values were calculated using Formula (2). Eutectic alloy Co75Si25 containing IMC composition Co_2_Si was the leader for almost all NO_3_RR synthesis potentials (Figure 5). In Figure 5a–c, diagrams with the resulting FE at all tested potentials for each sample of the electrocatalysts are presented. The summary graph in Figure 5d clearly shows the same FE values for all samples at the same time.

The sample containing the intermetallic compound Co_2_Si had the highest FE value, which reached 80.8% at a potential of −0.585 V. The replacement of silicon in the composition for iron (this was the sample Co75Fe25) led to the production of only a solid solution without the formation of IMC, also based on cobalt, and at the same time, the maximum achieved FE value was much lower and was equal to 63.0%, and with a no longer advantageous potential value of −0.685 V. The presence of 25 at.% of chromium (for the Co75Cr25 sample) led to an almost ***complete*** inhibition of the nitrate reduction process already at a potential below −0.385 V, and the maximum value reached did not exceed FE = 13.2% at E = 0.385 V.

The high value of the catalytic efficiency of the Co75Si25 alloy may have been due to the presence of IMC Co_2_Si in this alloy, and the high FE value is discussed in more detail in Section 2.4. “A brief summary of the elucidation for the proposed mechanism of electrocatalysis”.

The NH_3_ yield rate values calculated by Formula (3) are shown in the summary chart in Figure 5e. The maximum result was also achieved on the Co75Si25 sample. It is noteworthy that this value was achieved at a slightly higher potential than for the maximum FE. Apparently, the most effective value (optimum) of the potential for both FE and NH_3_ yield rate lay in the range from E = −0.585 to −0.685 V. According to Figure 5e, the highest rate of ammonia production was observed for the Co75Si25 sample, reaching a maximum value of 22.3 µmol h^−1^ cm^−2^ at E = −0.785 V, while the maximum value of FE was recorded at E = −0.585 V. The rate of ammonia production on the Co75Fe25 sample was two times lower than on Co75Si25 at the same potential.

### 2.3. Electrochemical Analysis of Electrocatalysts

#### 2.3.1. Determination of the Electrochemically Active Surface Area (ECSA)

**Figure 6:**
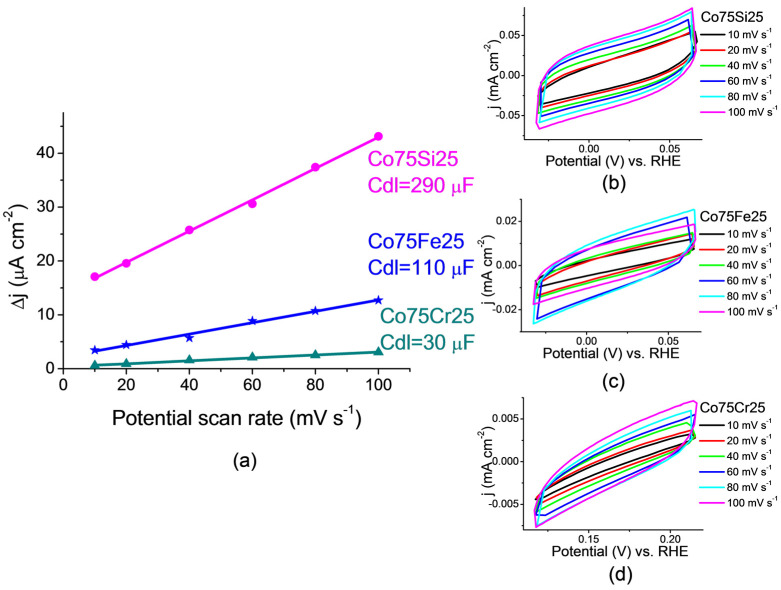
(**a**) The electrochemically active surface areas of the electrocatalyst samples presented as a double layer capacity. The series of cyclic voltammograms at scan rates of 10, 20, 40, 60, 80, and 100 mV s^−1^ for the samples: (**b**) Co75Si25; (**c**) Co75Fe25; (**d**) Co75Cr25.

The estimate of the electrochemically active surface area of the catalyst (ECSA) is presented in Figure 6. To obtain the C_dl_ values (double layer capacity) of all samples, a graph of the average current density was plotted depending on the scanning speed. ECSA was proportional to C_dl_.

The highest C_dl_ value was obtained for Co75Si25 (0.29 mF cm^−2^), and therefore, the surface of this catalyst had a large number of active sites that participated in the reduction of nitrate ions. Also, for this catalyst, FE was also the most important, and, consequently, the active centers of the catalyst were more selective for the conversion of NO_3_^−^ to NH_3_. The presence of chromium led to the fact that the Co75Cr25 catalyst with the lowest FE value had the smallest C_dl_ = 0.03 mF cm^−2^, which was 10 times less than that of Co75Si25 and 3.5 times less than that of Co75Fe25.

#### 2.3.2. Electrochemical Impedance Spectroscopy (EIS)

**Figure 7:**
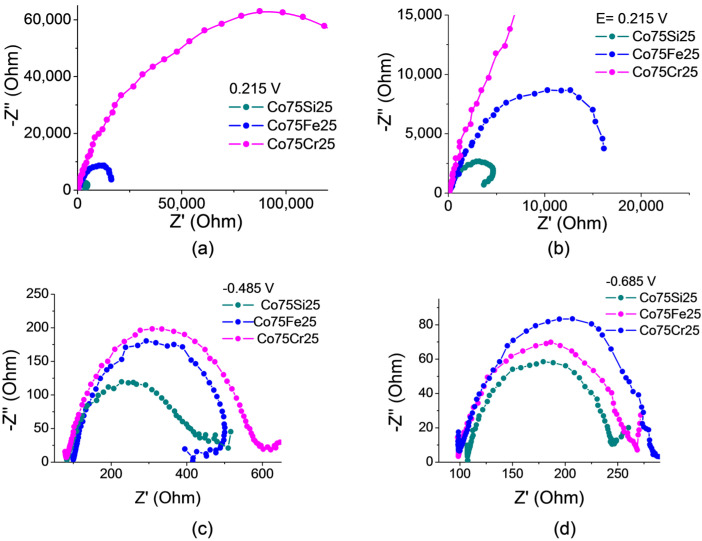
Nyquist curves for electrocatalyst samples Co75Si25, Co75Fe25, and Co75Cr25 in 12 mM NaNO_3_ with 0.05 M Na_2_SO_4_ at the following potentials (vs. RHE): (**a**) 0.215 V; (**b**) 0.215 V (for the enlarged fragment); (**c**) −0.485 V; (**d**) −0.685 V.

The radius of the Nyquist graphs is related to the resistance to charge transfer, and it is believed that a smaller radius indicates fast and efficient charge transfer at a potential (E = 0.215 V) in the non-Faraday region (Figure 7a,b). The graph shows that changes in the composition of the samples also changed the conductive properties of the electrodes.

The Co75Si25 sample contained, together with IMC, apparently more accessible catalytic centers, and the radius was the smallest among electrocatalysts, which indicates a low resistance to charge transfer. The Co75Fe25 sample had a higher resistance than the catalyst with IMC. The Co75Cr25 sample had a significant charge transfer resistance. A comparison of all samples suggests that the appearance of IMC promotes charge transfer at the cathode and increases the kinetics (rate) of NO_3_RR.

It is clearly noticeable that for the EIS spectra, the difference between the radius values for all samples decreased as the cathode potential of the spectrum measurement increased (Figure 7c for E = −0.485 V and Figure 7d for E = −0.685 V), which indicates an increase in the rate of charge transfer at the interface of the surface–electrolyte phases in NO_3_RR and side processes.

### 2.4. A Brief Summary of the Elucidation for the Proposed Mechanism of Electrocatalysis

Based on a significant pool of recent literature data on NO_3_RR, cobalt is an active catalytic component. Iron is also an active component that enhances the role of cobalt in the tandem effect (high adsorption of nitrate ions on iron and high kinetics of reduction of nitrate ions on cobalt). Chromium is an inhibitory component (presumably, the delayed kinetics of desorption of products from the surface). In this case, silicon is considered to be a neutral component. Based on these statements, it follows that the electrocatalyst Co75Cr25 should be significantly inferior to Co75Fe25, which was observed in the experiment (FE_max_ = 63.0% >> 13.2%). And here is an outstanding result for Co75Si25 compared to Co75Fe25 (FE_max_ = 80.8% > 63.0%), where the expected superiority of the tandem effect requires additional explanations.

First, the EIS and ECSA results provide evidence in favor of Co75Si25. The character of the catalytic centers and the rate of charge transfer are also confirmed by the FE and ammonium yield rate data. Second, Co75Si25 was the only one of the studied samples in which the IMC Co_2_Si was found.

IMCs in electrocatalytic reactions are currently comparatively poorly studied [17], and researchers mainly focus on IMCs with at least one of the components being a noble metal. It is noted that the advantage of electrocatalysts produced from IMC is the increased stability due to the existence of nanocrystals, but at the same time, the mechanism of reactions and the nature of active centers need to be clarified. The advantage is the ability to customize the electronic structure of the active centers and the well-controlled composition and structure of the IMC.

**Figure 8:**
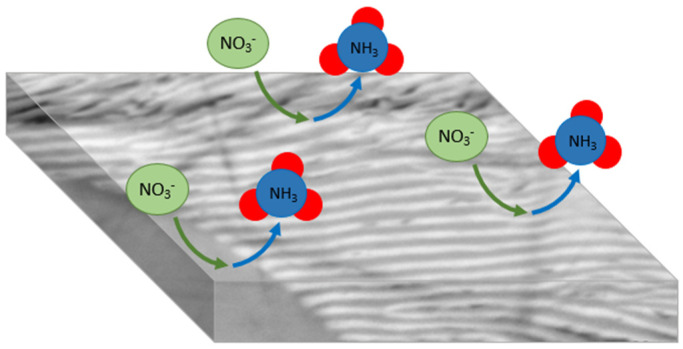
The general scheme of the proposed mechanism of nitrate ion reduction to ammonia on the surface of an electrocatalyst containing IMC.

It should also be mentioned that when talking about the catalytic activity of Co-electrocatalysts (and Fe- and Cr-electrocatalysts, too), they usually do not mean bare metal surfaces, but oxides; for example, it is believed that Co_3_O_4_ is much more active than CoO [33,34,35,36] in NO_3_RR due to the large number of dislocations and disorders/defects the crystal lattice, which leads to the formation of new catalytic centers. It should be noted that the search for direct synthesis strategies for intermetallic nanoarchitectures remains a difficult task. As noted recently [10], intermetallic nanoarchitectures (nanowires, nanosheets, etc.) often expose well-defined crystallographic facets, defects, and grain boundaries. These structural features of intermetallic nanoarchitectures lead to a difference in the binding strength of reaction intermediates and typical intermetallic nanoparticles, increasing the catalytic efficiency. A visual and simplified diagram of such a mechanism can be presented in Figure 8.

Thus, elucidation of the efficiency of IMCs as electrocatalysts in NO_3_RR requires further research, including computer calculations, while, according to the authors’ opinion, IMCs as electrocatalysts present not only a huge potential for future research and great optimism inherent in the search for effective catalysts for high-demanded processes such as NRR, NO_3_RR, NO_2_^−^RR, CO_2_RR, and electrochemical synthesis of urea through C-N coupling reactions, but also the ethanol oxidation electrocatalytic reaction [37].

## 3. Materials and Methods

### 3.1. Materials and Catalyst Preparation

Sodium nitrate (NaNO_3_, chemical purity) and sodium sulfate (Na_2_SO_4_, chemical purity) were used without additional purification. Distilled water was employed for all experiments.

Pieces of silicon (purity 99.99 wt.%), iron (99.95 wt.%), cobalt (99.95 wt.%), and chromium (99.95 wt.%) were used as the starting components. Syntheses of polycrystalline samples of Co75Si25; Co75Fe25; and Co75Cr25 (at.%) were carried out using an Edmund Bŭhler AM (Edmund Buchler GmbH, Heichingen, Germany) arc melting system with a water cooled copper hearth and a high-purity argon atmosphere with a Zr getter. The samples were remelted several times to reach homogeneity. The total mass losses after melting were smaller than 0.5%. The samples were then heat-treated at 800 °C in a vacuum quartz ampoule for 150 h.

For the electrochemical experiment, the alloy samples were cut, ground, polished, and then pressed into thermosetting plastics.

### 3.2. Catalyst Material Characterization

Scanning electron microscopy (SEM) analysis was performed with a LEO EVO 50 XVP electron microscope (Carl Zeiss, Jena, Germany) equipped with an INCA Energy 350 energy dispersion detector (EDX) (Oxford Instruments, UK), and optical microscopic images were taken with an inverted routine microscope for materials ZEISS Axio Vert.A1 (Zeiss AG, Jena, Germany).

The powder X-ray diffraction (PXRD) patterns were obtained with a STOE STADI P diffractometer (STOE & Cie GmbH: Darmstadt, Germany) equipped with a Ge-monochromator, CuK_α1_ radiation, λ = 1.54056 Ǻ, linear PSD in the transmission geometry. The samples were examined in the region 2θ = 10–90° with a scanning step of 0.01° and an exposure time of 30 s per point. The powder X-ray diffraction (PXRD) was carried out at room temperature using a DRON-3 diffractometer (St. Petersburg, Russia) (CuK_α_ 1.5418 Å, graphite monochromator, reflection geometry, angle range 20° < 2θ < 80°, the pitch of 0.05, counting time 10 s per point). The powders for PXRD were ground to a finely dispersed state in an agate mortar. The samples were identified by comparing theoretical and experimentally obtained X-ray diffraction patterns using a WinXPOW version 2.24.

### 3.3. Electrochemical Measurements

#### 3.3.1. Linear Voltammetry and Chronoamperometry

Electrochemical measurements were carried out at room temperature using an Autolab PGSTAT 302N potentiostat (Metrohm AG, Herisau, Switzerland) with a three-electrode cell and an Ag/AgCl electrode as a reference electrode. Electrocatalysts of the alloy samples were used as the working electrode, and a platinum plate was used as the counter-electrode. All potential values were recalculated vs. the reversible hydrogen electrode (RHE) according to the formula E_RHE_ = E _applied Ag/AgCl_ + 0.202 + 0.059 × pH, unless otherwise noted.

Linear voltammetry (LVs) at a potential scan rate of 50 mV s^−1^ was performed in a cathode–anode space-separated electrochemical cell with a total volume of 60 mL. Chronoamperometry electrochemical reactions of NO_3_RR were carried out for 1 h in the same cell. The electrolyte was a solution of 1000 ppm (12 mmol L^−1^) of NaNO_3_ in 0.05 M Na_2_SO_4_ degassed by an Ar flow before the tests.

#### 3.3.2. ECSA Evaluation

The ECSA value was calculated from the value of the double layer electrochemical capacitance (C_dl_) obtained by measuring CV (cyclic voltammogram) in the double layer potential range, i.e., the non-Faradaic area. All catalysts were scanned in the potential range from 0.165 V to 0.265 V vs. RHE in 12 mM NaNO_3_ with a 0.05 M Na_2_SO_4_ electrolyte at different scan rates (10 to 100 mV s^−1^). The values of the current density at 0.215 V vs. RHE at different scan rates were calculated, and the curves of the dependence of scan rates for each catalyst were plotted. The dependences of the current densities vs. the scan rates were obtained, and the C_dl_ values were found accordingly. ECSA was calculated as

(1) $$ECSA=\frac{C_{dl}}{C_{s}}\;,$$

where C_s_ = 0.4 F∙m^−2^ is the total specific capacitance for an atomically smooth planar surface under homogeneous electrolytic conditions.

#### 3.3.3. Impedance Response Testing

Impedance spectra were measured in a three-electrode undivided cell (60 mL) at room temperature in a solution of 12 mM of NaNO_3_ in 0.05 M Na_2_SO_4_. The Ag/AgCl reference electrode was used. The auxiliary electrode was a platinum plate. Measurements were carried out using a PS-20 potentiostat-galvanostat with an electrochemical impedance (EIS) measurement module FRA (SmartStat, Chernogolovka, Russia) in the frequency range from 100 kHz to 0.01 Hz at an AC voltage amplitude of 5 mV. The time of immersion of the sample corresponded to the time of impedance measurement without preliminary exposure in the medium.

### 3.4. Detection of Ammonia

**Figure 9:**
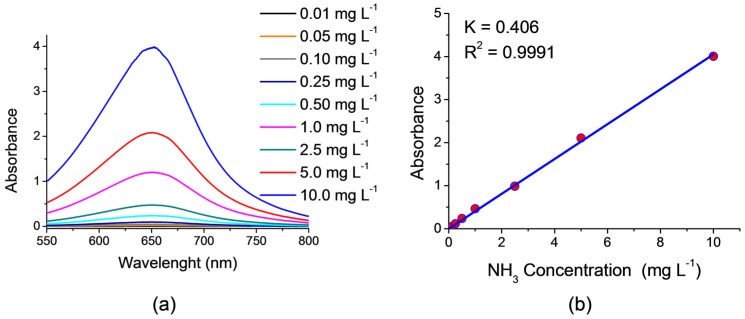
Detection of ammonia: (**a**) UV–VIS absorption spectra, and (**b**) calibration line for testing NH_3_.

The detection of the ammonia content after NO_3_RR was carried out using the indophenol method, according to the methodology provided from the reference below. UV–VIS absorption spectra (Figure 9a) were recorded using a Shimadzu 3600 Plus spectrophotometer (Shimadzu, Japan) in a standard 1 cm quartz cuvette. Two milliliters of 5 wt% sodium salicylate in 1.0 M NaOH were added to 2 mL of the tested solution, and then 1 mL of 0.05 M NaClO and Na_2_[Fe(NO)(CN)_5_] (0.2 mL, 1 wt%) were added. The solutions were kept at 40 °C for 1 h. The absorption maximum was observed at λ = 652 nm, for which a calibration graph was plotted. The resulting calibration graph is described by the equation (y = 0.406x; R^2^ = 0.9991) and showed a good linear relationship between the absorbance value and the NH_3_ concentration in the range from 0.01 mg mL^−1^ to 10 mg L^−1^ (Figure 9b).

Then, we calculated the Faradaic efficiency by the formula

(2) $$FE(NH_{3})=\frac{8\times F\times n(NH_{3})}{Q},$$

where n(NH_3_) denotes the amount (mol) of NH_3_; F is the Faradaic constant (96.485 C mol^−1^); Q is the total charge passed through the electrode; and 8 is the number of electron (n) transfers required to form 1 mol of ammonia.

The ammonia yield rate (yield) was defined as

(3) $$yield(NH_{3})=\frac{C(NH_{3})\times V}{17\times t\times S}$$

where C(NH_3_) denotes the mass concentration (μg mL^−1^) of NH_3_ calculated from the UV–VIS spectra; t is the electrolysis time; S is the geometric area of the working electrode (1 cm^2^); and V is the volume of the electrolyte.

## 4. Conclusions

A sustainable reaction of electrocatalytic conversion in ammonia production in ambient conditions is currently in high demand, as well as urgent research due to the real potential replacement of the environmentally unfavorable Haber–Bosch process. A series of electrocatalysts based on two-component cobalt alloys with cheap base metals was synthesized. Unexpectedly, the sample with an intermetallic compound of the composition Co_2_Si turned out to be the most highly efficient. The main results of the completed study are listed below:

- Using scanning electron microscopy and EDX, it was shown that the Co75Si25 alloy consists of two phases of different compositions, one of which belongs to the intermetallic compound Co_2_Si. The presence of Co, which has a catalytic effect in NO_3_RR in the intermetallic state, is of fundamental importance for this work, since it is able to enhance the electrocatalytic properties of the Co catalyst even more and to a significant extent.
- The XRD measurement results confirm that the Co75Si25 sample is a two-phase sample: one phase is the intermetallic compound Co_2_Si, the second one is a low-temperature Co phase. The Co-Fe and Co-Cr catalysts are solid solutions of the compositions Co75Fe25 and Co75Cr25, where the cobalt structure is preserved.
- Optical microscopic images of the surface of all catalyst samples also confirm well-defined phases (solid solution and IMC) only in the case of Co75Si25.
- Based on the results of linear voltammetric studies, seven potential values were selected, at which NO_3_RR was performed for 1 h for each sample of the electrocatalyst.
- As a result of NO_3_RR, an excellent volcanic dependence of the Faradaic efficiency and the ammonia yield rate vs. applied potential during chronoamperometry was obtained. The highest FE result of 80.8% at E = −0.585 V (RHE) and the ammonia yield rate of 22.3 µmol h^−1^ cm^−2^ at E = −0.685 V (RHE) indicate the progressive role of IMC as the main component of the electrocatalyst.
- The ECSA method shows that the highest C_dl_ value was obtained for Co75Si25 (209 µF cm^−2^), and therefore the surface of this catalyst has a large number of active sites involved in the process of nitrate ion reduction. According to EIS, it was found that the presence of IMC promotes charge transfer at the cathode and thereby increases the rate of the reaction of nitrate to ammonia conversion.

Thus, this study demonstrates the promise and enormous potential of IMC as the main component of highly efficient electrocatalysts for NO_3_RR. This work can serve primarily as a starting point for future studies of electrocatalytic conversion reactions in the production of ammonia using IMC catalysts based on non-noble metals.

## Figures and Tables

**Table 1:** The concentrations of elements in the alloys and the phase composition of the alloys.

Sample	State	Concentration of Elements in the Alloy, at. %			
		Co	Si	Fe	Cr
Co75Si25	eutectic alloy	67.45 ± 0.05	32.55 ± 0.05	-	-
	solid solution	74.5 ± 0.2	25.5 ± 0.2	-	-
Co75Fe25	solid solution	69.4 ± 0.1	-	30.6 ± 0.1	-
Co75Cr25	solid solution	74.7 ± 0.5	-	-	25.3 ± 0.5

## Data Availability

The original contributions presented in this study are included in the article. Further inquiries can be directed to the corresponding author.

## Abbreviations

The following abbreviations are used in this manuscript:

NO_3_RR	nitrate reduction reaction
NO_2_^−^RR	nitrite reduction reaction
CO_2_RR	CO_2_ reduction reaction
IMC	intermetallic compounds
FE	Faradaic efficiency
SEM	scanning electron microscopy
EDX	energy dispersion analysis
XRD	X-ray diffraction
RHE	reversible hydrogen electrode
LVs	linear voltammograms
CV	cyclic voltammetry
EIS	electrochemical impedance spectra
ECSA	electrochemically active surface area
C_dl_	double layer electrochemical capacitance

## References

[1] Jiang Z., Wang Y., Lin Z., Yuan Y., Zhang X., Tang Y., Wang H., Li H., Jin C., Liang Y. (2023). Molecular Electrocatalysts for Rapid and Selective Reduction of Nitrogenous Waste to Ammonia. Energy Environ. Sci..

[2] Ma C., Zhang H., Xia J., Zhu X., Qu K., Feng F., Han S., He C., Ma X., Lin G. (2024). Screening of Intermetallic Compounds Based on Intermediate Adsorption Equilibrium for Electrocatalytic Nitrate Reduction to Ammonia. J. Am. Chem. Soc..

[3] Huang Y., He C., Cheng C., Han S., He M., Wang Y., Meng N., Zhang B., Lu Q., Yu Y. (2023). Pulsed Electroreduction of Low-Concentration Nitrate to Ammonia. Nat. Commun..

[4] Murphy E., Liu Y., Matanovic I., Rüscher M., Huang Y., Ly A., Guo S., Zang W., Yan X., Martini A. (2023). Elucidating Electrochemical Nitrate and Nitrite Reduction over Atomically-Dispersed Transition Metal Sites. Nat. Commun..

[5] Anastasiadou D., Van Beek Y., Hensen E.J.M., Costa Figueiredo M. (2023). Ammonia Electrocatalytic Synthesis from Nitrate. Electrochem. Sci. Adv..

[6] Kuznetsova I., Lebedeva O., Kultin D., Mashkin M., Kalmykov K., Kustov L. (2024). Enhancing Efficiency of Nitrate Reduction to Ammonia by Fe and Co Nanoparticle-Based Bimetallic Electrocatalyst. Int. J. Mol. Sci..

[7] Wu Q., Zhu W., Ma D., Liang C., Wang Z., Liang H. (2024). Screening of Transition Metal Oxides for Electrocatalytic Nitrate Reduction to Ammonia at Large Currents. Nano Res..

[8] Shankar A., Marimuthu S., Maduraiveeran G. (2023). Heterostructured Iron-Cobalt Sulfides Nanoclusters Entrenched in 3D-Nanosheets as High-Efficient Electrocatalysts for Oxygen Evolution Reaction. Int. J. Hydrogen Energy.

[9] Zhou J., Gao S., Hu G. (2024). Recent Progress and Perspectives on Transition Metal-Based Electrocatalysts for Efficient Nitrate Reduction. Energy Fuels.

[10] Kim H.Y., Jun M., Joo S.H., Lee K. (2023). Intermetallic Nanoarchitectures for Efficient Electrocatalysis. ACS Nanosci. Au.

[11] Zhang Q., Song M., Luo G., Shen T., Hu H., Wang D. (2024). Recent Advances of High-Entropy Intermetallics for Electrocatalysis. Chem. Mater..

[12] Nakaya Y., Furukawa S. (2024). High-Entropy Intermetallics: Emerging Inorganic Materials for Designing High-Performance Catalysts. Chem. Sci..

[13] Xiao W., Yan D., Zhao Q., Bukhvalov D., Yang X. (2024). Regulating Electrocatalytic Properties of Oxygen Reduction Reaction via Strong Coupling Effects between Co-NC Sites and Intermetallic Pt3Co. Appl. Catal. B.

[14] Lin F., Li M., Zeng L., Luo M., Guo S. (2023). Intermetallic Nanocrystals for Fuel-Cells-Based Electrocatalysis. Chem. Rev..

[15] Wu L., Li L., Wang H., Mo X., Zhang H., Zhang Q., Xiao Y., Kang J., He Y. (2024). Porous Ni3Si Intermetallics as Electrocatalysts for Hydrogen Evolution Reaction. Int. J. Hydrogen Energy.

[16] Walter C., Menezes P.W., Driess M. (2021). Perspective on Intermetallics towards Efficient Electrocatalytic Water-Splitting. Chem. Sci..

[17] Liu J., Lee C., Hu Y., Liang Z., Ji R., Soo X.Y.D., Zhu Q., Yan Q. (2023). Recent Progress in Intermetallic Nanocrystals for Electrocatalysis: From Binary to Ternary to High-entropy Intermetallics. SmartMat.

[18] Xie M., Tang S., Li Z., Wang M., Jin Z., Li P., Zhan X., Zhou H., Yu G. (2023). Intermetallic Single-Atom Alloy In–Pd Bimetallene for Neutral Electrosynthesis of Ammonia from Nitrate. J. Am. Chem. Soc..

[19] Wei H., Tang C., Wang Y., Feng Y., Shi H., Cui M., Liu H., Dou S., Li L. Intermetallic Compounds for Nitrogen Electrochemistry. Green Energy Environ..

[20] Lan J., Wang Z., Kao C., Lu Y.-R., Xie F., Tan Y. (2024). Isolating Cu-Zn Active-Sites in Ordered Intermetallics to Enhance Nitrite-to-Ammonia Electroreduction. Nat. Commun..

[21] Gerke C.S., Foley G.D.Y., Wilder L.M., Yang Y., Young J.L., Bedford N.M., Miller E.M., Thoi V.S. (2025). Conformal Electrochemical Deposition of Intermetallic AuCu Thin Films for Convergent C–N Coupling. J. Mater. Chem. A.

[22] Xiao Y., Tan X., Du B., Guo Y., He W., Cui H., Wang C. (2024). Strained Au Skin on Mesoporous Intermetallic AuCu_3_ Nanocoral for Electrocatalytic Conversion of Nitrate to Ammonia across a Wide Concentration Range. Angew. Chem..

[23] Gao Q., Yao B., Pillai H.S., Zang W., Han X., Liu Y., Yu S.-W., Yan Z., Min B., Zhang S. (2023). Synthesis of Core/Shell Nanocrystals with Ordered Intermetallic Single-Atom Alloy Layers for Nitrate Electroreduction to Ammonia. Nat. Synth..

[24] Wang P., Liu C., Rao L., Tao W., Huang R., Huang P., Zhou G. (2024). Transient Heating Synthesis of a Highly Ordered Ga–Cu Intermetallic Antiperovskite for Efficient Ammonia Electrosynthesis and Ultrastable Zinc–Nitrate Fuel Cells. Energy Environ. Sci..

[25] Ge J., Wei T., Ding J., Wang Z., Liu Q., Qi G., Hu G., Luo J., Liu X. (2023). Al-Ce Intermetallic Phase for Ambient High-Performance Electrocatalytic Reduction of Nitrate to Ammonia. ChemCatChem.

[26] Zhang H., Ma C., Wang Y., Zhu X., Qu K., Ma X., He C., Han S., Liu A., Wang Q. (2024). Transition Metal-Gallium Intermetallic Compounds with Tailored Active Site Configurations for Electrochemical Ammonia Synthesis. Angew. Chem. Int. Ed..

[27] Zhang J., Lan J., Xie F., Luo M., Peng M., Palaniyandy N., Tan Y. (2024). Nanoporous Copper Titanium Tin (Np-Cu2TiSn) Heusler Alloy Prepared by Dealloying-Induced Phase Transformation for Electrocatalytic Nitrate Reduction to Ammonia. J. Colloid Interface Sci..

[28] Lodaya K.M., Tang B.Y., Bisbey R.P., Weng S., Westendorff K.S., Toh W.L., Ryu J., Román-Leshkov Y., Surendranath Y. (2024). An Electrochemical Approach for Designing Thermochemical Bimetallic Nitrate Hydrogenation Catalysts. Nat. Catal..

[29] Hou Z., Hua M., Liu Y., Deng J., Zhou X., Feng Y., Li Y., Dai H. (2024). Exploring Intermetallic Compounds: Properties and Applications in Catalysis. Catalysts.

[30] Zhang K., Liu Y., Pan Z., Xia Q., Huo X., Esan O.C., Zhang X., An L. (2024). Cu-Based Catalysts for Electrocatalytic Nitrate Reduction to Ammonia: Fundamentals and Recent Advances. EES Catal..

[31] Miller D.M., Liu M.J., Abels K., Kogler A., Williams K.S., Tarpeh W.A. (2024). Engineering a Molecular Electrocatalytic System for Energy-Efficient Ammonia Production from Wastewater Nitrate. Energy Environ. Sci..

[32] Lebedeva O., Zakharov V., Kuznetsova I., Kultin D., Kustov L., Aslanov L., Chernyshev V., Savilov S., Dunaev S., Kalmykov K. (2024). Green Synthesis of the Triazine Derivatives and Their Application for the Benign Electrocatalytic Reaction of Nitrate Reduction to Ammonia. Chem. A Eur. J..

[33] Ye M., Jiang X., Zhang Y., Liu Y., Liu Y., Zhao L. (2024). Enhanced Electrocatalytic Nitrate Reduction to Ammonia Using Functionalized Multi-Walled Carbon Nanotube-Supported Cobalt Catalyst. Nanomaterials.

[34] Wang J., Cai C., Wang Y., Yang X., Wu D., Zhu Y., Li M., Gu M., Shao M. (2021). Electrocatalytic Reduction of Nitrate to Ammonia on Low-Cost Ultrathin CoO *_x_* Nanosheets. ACS Catal..

[35] Zhao F., Hai G., Li X., Jiang Z., Wang H. (2023). Enhanced Electrocatalytic Nitrate Reduction to Ammonia on Cobalt Oxide Nanosheets via Multiscale Defect Modulation. Chem. Eng. J..

[36] Meng X., Tan X., Ma Y., Obisanya A.A., Wang J., Xiao Z., Wang D. (2024). Recent Progress in Cobalt-Based Electrocatalysts for Efficient Electrochemical Nitrate Reduction Reaction. Adv. Funct. Mater..

[37] Wang Y., Meng H., Yu R., Hong J., Zhang Y., Xia Z., Wang Y. (2025). Unconventional Interconnected High-Entropy Alloy Nanodendrites for Remarkably Efficient C−C Bond Cleavage toward Complete Ethanol Oxidation. Angew. Chem..

